# Exploring ionic liquid assisted chemical bath deposition of a highly uniform and transparent cadmium sulfide thin film for photovoltaic applications

**DOI:** 10.1039/d4ra06320a

**Published:** 2025-02-13

**Authors:** Taskina Nasrin, Vidhya Selvanathan, Md. Ariful Islam, Md. Mahfuzul Haque, Ayesha Wasima Rashid, Norasikin Ahmad Ludin, Puvaneswaran Chelvanathan, Tiong Sieh Kiong, Abdulaziz M. Alanazi, Hamad AlMohamadi, Ishtiaque M. Sayed, Md. Shahiduzzaman, Takashi Suemasu, Md. Akhtaruzzaman

**Affiliations:** a Solar Energy Research Institute (SERI), Universiti Kebangsaan Malaysia (UKM) 43600 Bangi Selangor Malaysia sheekeen@ukm.edu.my; b Institute of Sustainable Energy, Universiti Tenaga Nasional, Jalan Ikram-Uniten Kajang 43000 Selangor Malaysia vidhya@uniten.edu.my; c Institute of Advanced Research and Technology (IART), Dhaka Solar Ltd Dhaka-1212 Bangladesh; d Centre for Advanced Research in Sciences (CARS), University of Dhaka Dhaka 1000 Bangladesh; e Department of Mathematical and Physical Sciences, Faculty of Sciences and Engineering, East West University Dhaka-1212 Bangladesh; f Institute of Energy, University of Dhaka Dhaka 1000 Bangladesh; g Department of Chemistry, Faculty of Science, Islamic University of Madinah 42351 Madinah Saudi Arabia; h Department of Chemical Engineering, Faculty of Engineering, Islamic University of Madinah 42351 Madinah Saudi Arabia makhtar@iu.edu.sa; i Department of Physics, University of Dhaka Dhaka 1000 Bangladesh; j Nanomaterials Research Institute, Kanazawa University Kakuma Kanazawa 920-1192 Japan; k Institute of Applied Physics, University of Tsukuba 1-1-1 Tennodai Tsukuba Ibaraki 305-8573 Japan; l Sustainable Research Center, Islamic University of Madinah Madinah 42351 Saudi Arabia

## Abstract

Cadmium sulfide (CdS) is one of the most important semiconductor materials in solar cells. In this study, different concentrations (0–0.118 M) of 1-butyl-3-methylimidazolium tetrafluoroborate (BMIMBF_4_) ionic liquid (IL) are introduced as a novel complexing agent in dilute chemical bath deposition of CdS thin films. To comprehend the effectiveness of different ionic liquid concentrations as the complexing agent, the structural, morphological, electrical, and optoelectronic properties of the films were investigated. X-ray diffractogram of the CdS thin film exhibited peaks attributed to wurtzite structure, with peak intensity enhanced dramatically after IL addition. From morphological studies, a pinhole-free and uniformly deposited CdS film with large grain size was observed upon inclusion of 0.069 M IL. Optical characterization has shown good transparency up to 85% from the UV-vis spectroscopy analysis. With the variation of the ionic liquid concentration, there was no major difference observed in the energy bandgap. However, an increment in carrier concentration and reduction in resistivity of the deposited thin films were observed. The film with 0.069 M IL showed the maximum carrier concentration value of 7.51 × 10^14^ cm^−3^ with the lowest resistivity. Incorporating the optoelectronic properties of the deposited CdS films, numerical simulations were performed to validate those as electron transport layers for perovskite solar cells with the device structure of FTO/CdS (CdS-0 to CdS-3)/CsSnBr_3_/P3HT/Ag. Simulation results demonstrated that the fabricated CdS thin film fabricated with 0.069 M BMIMBF_4_ would be a promising candidate in perovskite solar cells with an efficiency of around 16.5%.

## Abstract 2

Improved cadmium sulfied (CdS) thin films *via* controlled deposition using ionic liquids.

## Graphical abstract

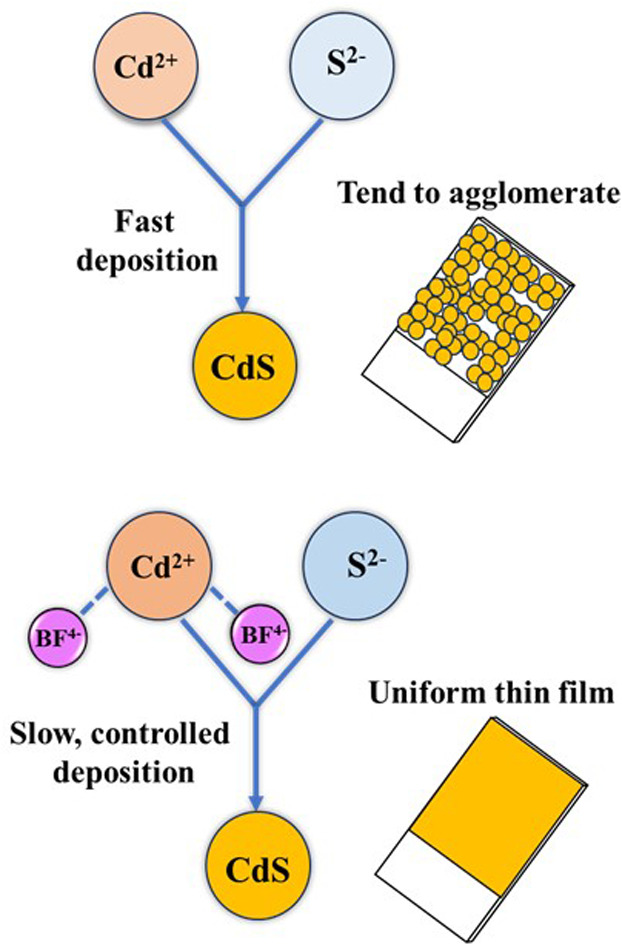

## 1. Introduction

The CdS is one of the low-cost materials which has excellent optoelectronic properties along with good lattice-matching properties, low resistivity, easy ohmic contact, and low interface defects.^1,2^ It is a compound of the group II–VI, an n-type semiconductor with an energy gap of ∼2.45 eV. Due to its easy fabrication process, this material is mostly used as window material in thin film photovoltaic solar cells (*e.g.* CdTe, Cu(In,Ga)Se_2_, Cu_2_ZnSnS_4_).^3,4^ The most popular techniques for depositing CdS thin films include spray pyrolysis, close-spaced sublimation, thermal evaporation, chemical bath deposition (CBD), and metal–organic chemical vapor deposition (MOCVD).^5^ Among all these deposition methods, CBD is especially favored due to its simplicity and cost-effectiveness. This method is a well-established technique for fabricating high-quality CdS thin films, and its fundamental principles have been extensively documented in the literature. Foundational studies by Lokhande,^5^ Ortega-Borges and Lincot^6^ have established the theoretical and experimental frameworks for CBD as a reliable deposition method, particularly for CdS thin films. These works provide detailed insights into the reaction mechanisms, kinetics, and thermodynamics of the deposition process. Building on these foundations, subsequent researchers – including Paul O'Brien and John McAleese,^7^ Y.-J. Chang *et al.*,^8^ and M. S. Aida & S. Hariech^9^ have expanded CBD's scope, addressing both key challenges and emerging opportunities in thin-film fabrication. Collectively, these studies serve as the cornerstone for understanding CBD's applicability and offer critical insights into optimizing parameters for advanced material development. By adjusting the bath parameters, such as duration, temperature, or the addition of other chemicals, the optoelectronic properties of the formed thin films can be modified.

The addition of a complexing agent is another crucial factor that is used to govern the rate of thin film deposition. Typically, the complexing agent binds with the metal ions in the solution to form complex ions and prevents immediate immersion deposition. Hence, the complexing agent's main role is to engage with the metal cations, which helps to slow down their reaction and stop the final product from precipitating in bulk amounts. Without any complexing agents, therefore, little to no film formation is anticipated since the product precipitates in the bulk solution rather than depositing properly on the substrate.^10^

Researchers have used several complexing agents (*e.g.*, ammonia, EDTA, acetylacetone, TEA, nitrilotriacetic acid, amino acid) in the CBD solution to alter the properties of the CdS thin films and studied the variation.^11,12^ For instance, V. D. Moreno-Regino *et al.* examined the structural, electrical, and optical properties of the films along with the presence of different organic compounds in the films, which were deposited by varying the ammonium hydroxide's (complexing agent) concentration in the bath solution. They found lower defects with higher uniformity in the film prepared with a 0.24 M concentration of the complexing agent, showing the best structural, electrical and optical properties.^12^ Arun Kumar *et al.* prepared hierarchical nanoflake-structured CdS thin films with different complexing agents using the CBD method at two deposition intervals. Ammonia, ammonia with triethanolamine or ethylene-diamine-tetra-acetic acid were used as complexing agents. The cubic phase of the deposited films was confirmed by X-ray diffraction. Further, they observed the increment of crystallite size in the deposited thin films for higher deposition time.^13^

Ionic liquids (ILs) are liquids at low temperatures (<100 °C) consisting of organic cations that are large in size and asymmetric (*i.e.* pyridinium, phosphonium, imidazolium) and inorganic or organic anions. The structure of these cations and anions as well as their interactions control the properties of ILs. By varying the combination and structural design of ions, the chemical and physical properties of ILs can be adjusted to a large range. In the electrolyte system, ILs were introduced as well stable solvents for stability improvement of dye synthesized solar cells (DSSCs).^14^ Besides, they play a significant role in energy level adjustment, surface defects modification, and homogenous nucleation, affecting charge transportation mechanism and the kinetics of crystal growth.

**Fig. 1:**
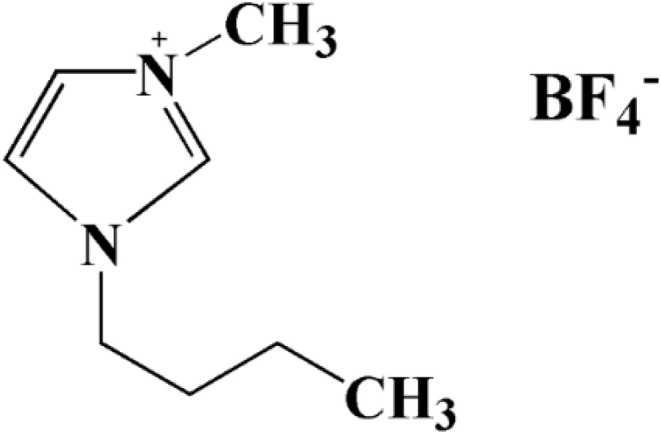
Chemical structure of BMIMBF_4_.

Some of the typical complexing agents used for the formation of CdS thin film *via* CBD are triethanolamine, ethanolamine and sodium citrate. Although using ionic liquids as the alternative complexing agent may not directly decrease the cost of material, these ionic liquids offer a promising approach to enhance the efficiency and environmental sustainability of thin film deposition processes. The unique properties of ionic liquids may justify the higher material cost by enabling higher-quality thin film deposition and reducing environmental impact.^15^ Moreover, ILs are known for their easy recycling processes, hence allowing the possibility of reusing them that reduces the overall production costs.^16^ Among several ILs, 1-butyl-3-methylimidazolium tetrafluoroborate (BMIMBF_4_) is well-known one that consists of electron-donating alkyl chains and electron-rich nitrogen atoms. Fig. 1 shows the chemical structure of this IL.

Though the use of IL is not a new thing for developing thin films for solar cells, to the best of our knowledge none reported on the incorporation of it in the CBD method to improve the optoelectronic properties of CdS thin-film that can be used in perovskite solar cells (PSCs). In this study, the BMIMBF_4_ IL was introduced as a complexing agent in the CBD method to improve the optical, electrical, and morphological properties for CdS thin film. As the properties of the deposited films suggested that they can be used as an electron transport layer (ETL) in PSC, a theoretical study on a PSC incorporating deposited CdS films as ETL was conducted by SCAPS-1D simulation package. To the best of our knowledge, no prior work has incorporated BMIMBF_4_ in the CBD process for CdS thin-film deposition, making this a pioneering study in leveraging the unique properties of ionic liquids for photovoltaic applications.

## 2. Methodology

### 2.1. Materials and methods

Cadmium sulphate (CdSO_4_), ammonium hydroxide (NH_4_OH), thiourea, and BMIMBF_4_ were obtained from Sigma Aldrich and utilized as raw materials without any kind of further treatment. CdSO_4_ and thiourea were dissolved in deionized water (DI water) to prepare the precursor solution that was kept stirring until it became an aqueous solution. Then CBD method was used to deposit CdS films on the soda-lime glass (SLG) substrate.

### 2.2. Fabrication of CdS thin films

CdS thin films were deposited on SLG substrates through the CBD method. At first, all substrates were cleaned with soap and then cleaned in chronological order with methanol–acetone–DI water in an ultrasonic cleaning system. Then glass substrates were dried with N_2_ gas, attached to the holder, and put on the growth beaker for deposition. A growth beaker consisting of 0.002 M of CdSO_4_, 1.31 M of NH_4_OH, 0.05 M of *N*-methyl thiourea were taken in a growth beaker in which BMIMBF_4_ concentration varied from 0 to 0.118 M. The considered concentrations of BMIMBF_4_ to produce different CdS films are tabulated in Table 1 along with the designation of the deposited films below. The deposition was performed at 80 °C for 50 minutes. When the deposition was completed, the samples were rinsed in 30% diluted ammonia followed by hot and room temperature DI water.

**Table 1:** Concentration of ionic liquid to deposit different CdS films

Designation	Amount of IL (M)
CdS-0	0
CdS-1	0.035
CdS-2	0.069
CdS-3	0.118

### 2.3. Waste disposal and management

The CBD solutions were carefully managed to prevent environmental contamination, particularly those containing BMIMBF_4_ and cadmium salts. Cadmium sulfide was safely precipitated using sodium sulfide and subsequently disposed of as hazardous waste. BMIMBF_4_ was recovered through phase separation and purification for reuse. Residual waste was neutralized and handled in strict compliance with approved hazardous waste disposal protocols and local environmental regulations.

### 2.4. Characterization

Structural properties, including the orientation of crystals and some other crystallographic properties, were assessed at room temperature by BRUKER aXSD8 Advance diffractometer with varying angles from 10° to 80° with 0.02° step size using Cu Kα radiation of 1.5408 Å wavelength. Raman spectroscopy (Thermo Scientific, Model: DXR2xi) was utilized to record the Raman spectra of the deposited thin films within the range of 100 cm^−1^ to 800 cm^−1^. A 532 nm laser was employed, where its power was kept below 5 mW to prevent laser-induced changes in the films. The surface morphology of the films including the cross-sectional view and elemental composition were examined by using field emission scanning electron microscopy (FESEM) of model Hitachi SU1510 and Ultra Dry EDS detector of Thermo Fisher Scientific. Lambda 900 UV/vis/NIR spectrophotometer was used to measure the transmittance and absorbance spectra of the films. The electrical properties of the deposited films were acquired by a Hall effect measurement system of model HMS ECOPIA 3000.

### 2.5. Numerical simulation

To validate the proper use of deposited CdS thin films as ETL in perovskite solar cell (PSC), SCAPS-1D software was used for simulating the solar cell. Poisson's equation, continuity equation of hole and electron are mainly solved by this software until they converge and different cell performance parameters are derived from the simulation.^17–19^ These equations are mentioned below.^20–24^

Continuity equation for electrons and holes, respectively:1
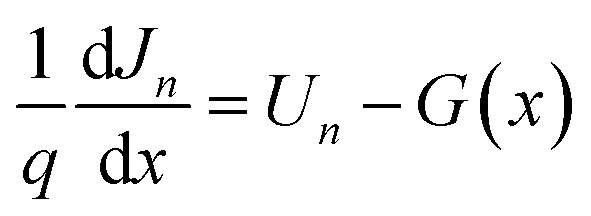
2
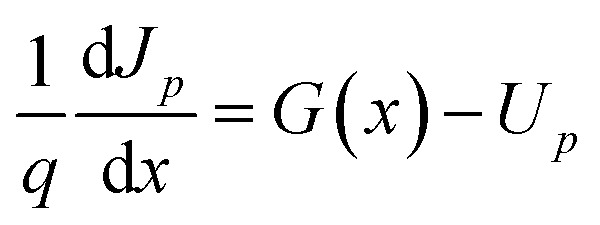
where, *q* is the elementary charge, *U*_*n*_ and *U*_*p*_ are recombination rates of electrons and holes, respectively, *J*_*n*_ and *J*_*p*_ are current densities of electrons and holes, respectively, and *G*(*x*) is the electron–hole pair generation rate at some distance *x* from the surface of the cell.

Poisson's equation:3
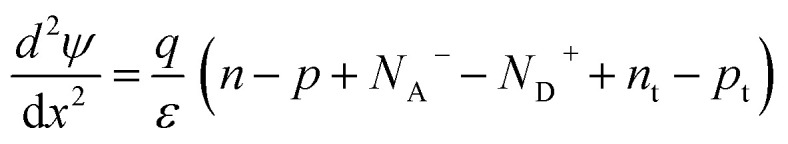
where, *Ψ* is electrostatic potential, *p* and *n* are the density of holes and electrons, respectively, *N*_D_^+^ and *N*_A_^−^ = concentration of ionized donors and acceptors, respectively, *n*_t_ and *p*_t_ are the concentration of trapped electrons and holes, respectively, and *ε* is material's permittivity.

## 3. Results and discussion

### 3.1. Structural properties

**Fig. 2:**
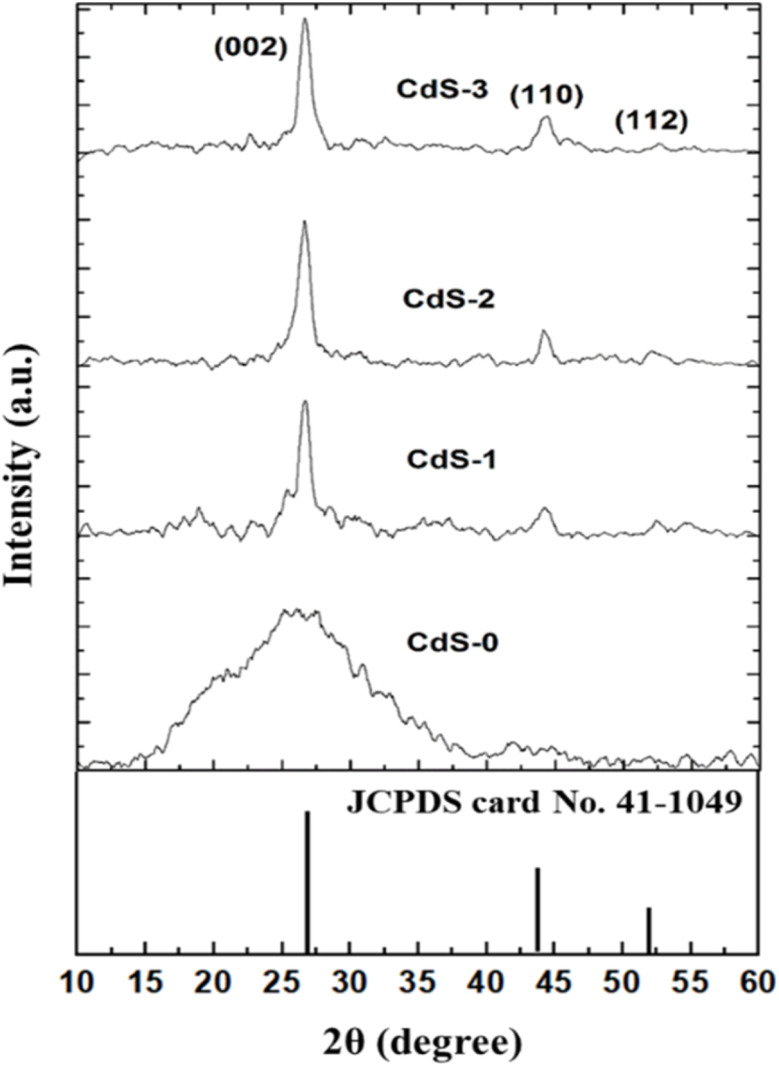
XRD pattern of deposited CdS thin film for increasing ionic liquid concentration.

X-ray diffraction patterns of the deposited CdS thin films for different concentrations of the IL are shown in Fig. 2. According to the literature, CdS crystals have two major crystalline phases – hexagonal and cubic. All the CdS thin films showed the CdS peak as a hexagonal structure which matches with the standard data from the JCPDS card (no. 41-1049).^3^ Bare CdS thin film showed a regular peak at 2*θ* = 26.7°, which corresponds to the (002) plane of the hexagonal form of CdS crystals. When the IL was introduced, the bare sample's amorphous characteristics changed, and its peak became more intense. The sharpest peak, which was observed for 0.069 M of IL, suggested that the film's level of crystallinity was increasing. Further, after adding IL, two small peaks became visible in the (100) and (112) planes. These two planes which corresponded to 2*θ* = 43.71° (110) and 51.85° (112), also matched with CdS phases. Increment of peak intensity due to the variation of ionic liquid concentration indicated the transition from amorphous to crystalline.

The intensity of the prominent peak increased with IL concentration which corresponds to increasing crystallite size as calculated using Debye–Scherrer's formula,^25^4
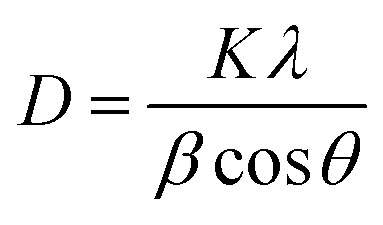
where, *K* is 0.89, *D* represents the average crystallite size, *λ* and *θ* are the light wavelength and Bragg's angle, respectively and *β* symbolizes the full width at a half maximum.

Introducing the IL increased the crystallinity dramatically and the increment of IL concentration caused an increase in crystallite size gradually, as shown in Table 2. (Eqn (5)) was used to calculate the average strain (*ε*) of the deposited thin films was calculated by using the Williamson–Hall equation as below.^25,26^5
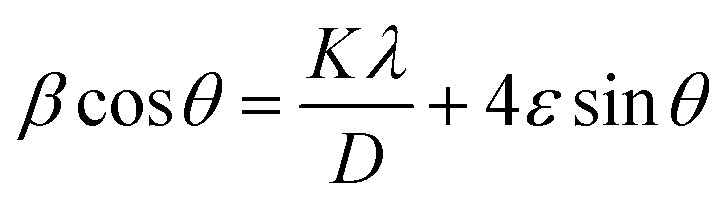


**Table 2:** Crystallite size, FWHM, strain and dislocation density

Sample	FWHM, *β* (degrees)	Strain, *ε*	Crystallite size, *D* nm	Dislocation density, *δ* (× 10^13^ cm^−2^)
CdS-0	14.214	0.267	0.567	31.06
CdS-1	1.238	0.023	6.519	0.235
CdS-2	1.213	0.022	6.656	0.226
CdS-3	1.085	0.019	7.441	0.181

Moreover, eqn (6) was used to calculate the dislocation density (*δ*) that is found due to the creation of imperfections in crystal orientation.^27,28^6
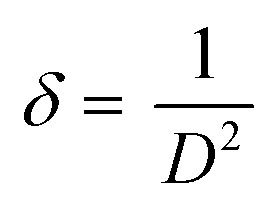


Table 2 also depicts the variation in strain and crystallite size. The lower value of strain signifies higher crystallinity.^29^ As the concentration of IL increases, the strain value decreases. The lowest and highest avg. strain (*ε*) value for IL assisted-CdS thin films were recorded as 0.019, and 0.023, respectively.

### 3.2. Raman spectrum analysis

**Fig. 3:**
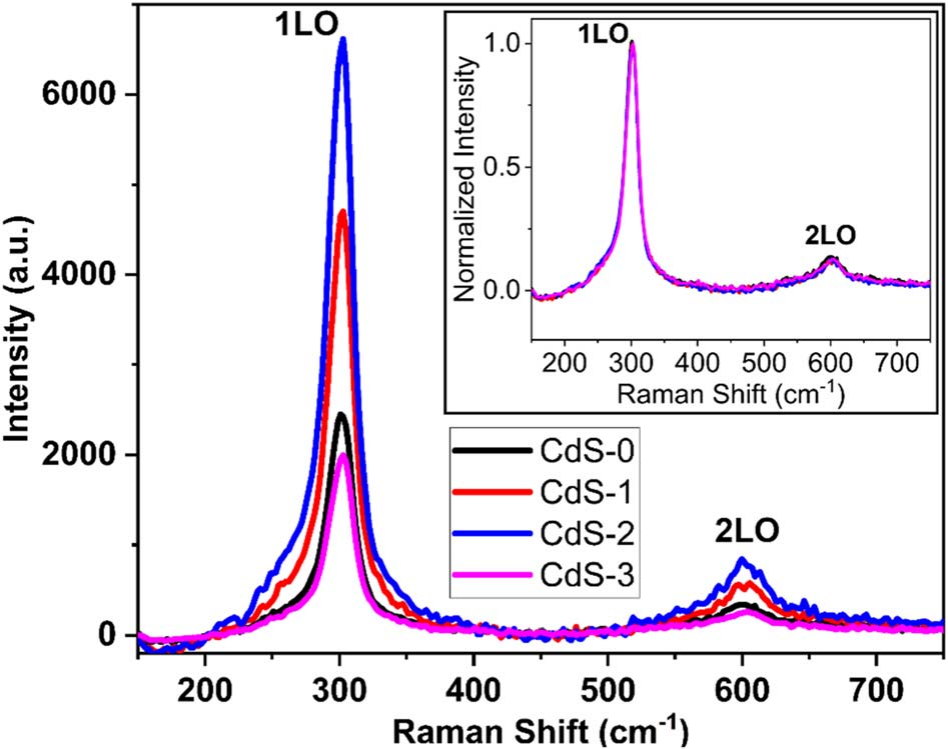
Raman spectra curve of deposited CdS thin films.

The structural characteristics of the deposited CdS thin films were investigated further at ambient conditions by Raman spectrum analysis, which are illustrated in Fig. 3. Analyzing these spectra, it has been found that, the progression in the longitudinal optical (LO) phonon mode dominates the spectra.^30^ Two peaks are found at 301.97 and 601.84 cm^−1^ in each spectrum, the first one is the A1 longitudinal optical (1LO) mode, and the other (weaker one) is the LO mode's overtone (2LO), which is consistent with earlier findings.^31–33^ The peak intensity of the deposited thin films may depend on several factors, such as crystallinity, interface stress, or the mode of surface phonon,^34^ amount of material, dimensions of the investigated area, laser power, *etc.* For deposited CdS thin films, it has been observed that CdS-2 has a higher peak intensity as compared to others. This may be due to the better crystallinity resulting in lower defects in the film.^35,36^ Moreover, with the ionic liquid concentration variation, peak positions in A1 longitudinal mode are shifted a little because of the phonon confinement effect.^33^ Earlier findings have also reported a shift in peak positions in the Raman spectrums for CdS thin films.^37,38^ For a clearer comparison, the normalized Raman spectra are presented as an inset in Fig. 3, allowing for the calculation of FWHM for the stronger peaks (1LO). Despite the observed variations in peak intensities, the FWHM values, found to be nearly equal (∼23.14 cm^−1^) across all films, suggest similar structural properties and homogeneity despite the observed differences in peak intensities. This demonstrates the complexity of the relationship between peak intensity and structural characteristics, indicating that while peak intensity reflects variations in crystallinity and defect levels, the consistent FWHM values point to a uniformity in the films' overall structural quality.

### 3.3. Morphological properties

**Fig. 4:**
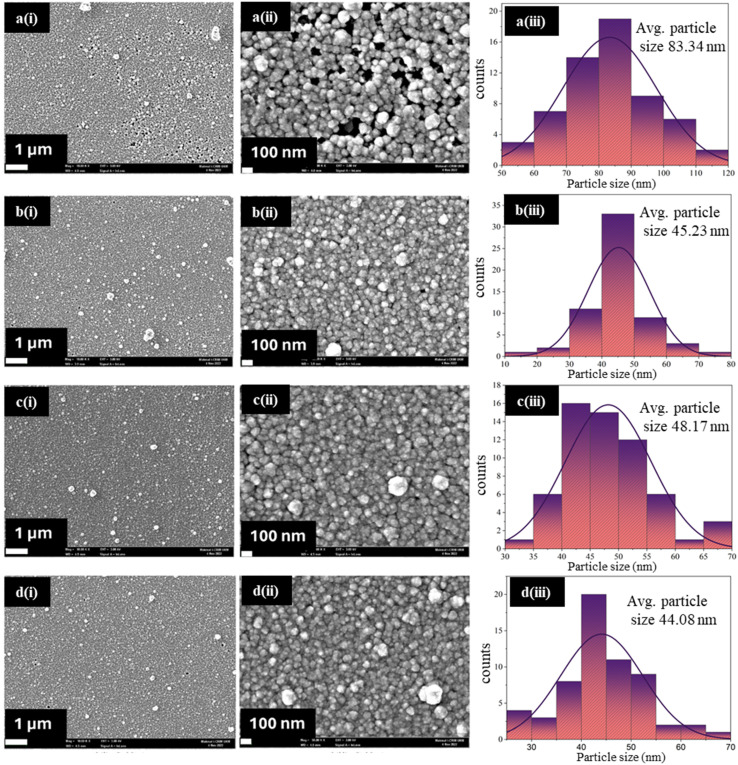
FESEM images of (a) CdS-0, (b) CdS-1, (c) CdS-2 and (d) CdS-3 films with different scale bars (1 μm and 100 nm) and size distribution histogram.

Fig. 4 depicts the FESEM images and particle size distribution of the CdS thin films. With the addition of different concentrations of IL, the films showed varying results in surface morphology compared to the bare CdS. No agglomeration was observed for CdS-0, but there were many pinholes that made the film non-uniform. Upon the addition of IL, the surface quality of the films improved up to CdS-2, with fewer pinholes and more compact packing. However, CdS-2 exhibited an average particle size of 48.17 nm, which is smaller than that of CdS-0 (83.34 nm), and agglomerates were observed. For CdS-3, the agglomerates were reduced, and the average particle size further decreased to 44.08 nm, but the surface exhibited signs of overgrowth, reducing the film uniformity. The improved surface morphology of CdS-2, with larger grain sizes and fewer pinholes can be attributed to the balanced reaction process facilitated by the IL, which allowed for better film coverage.

**Fig. 5:**
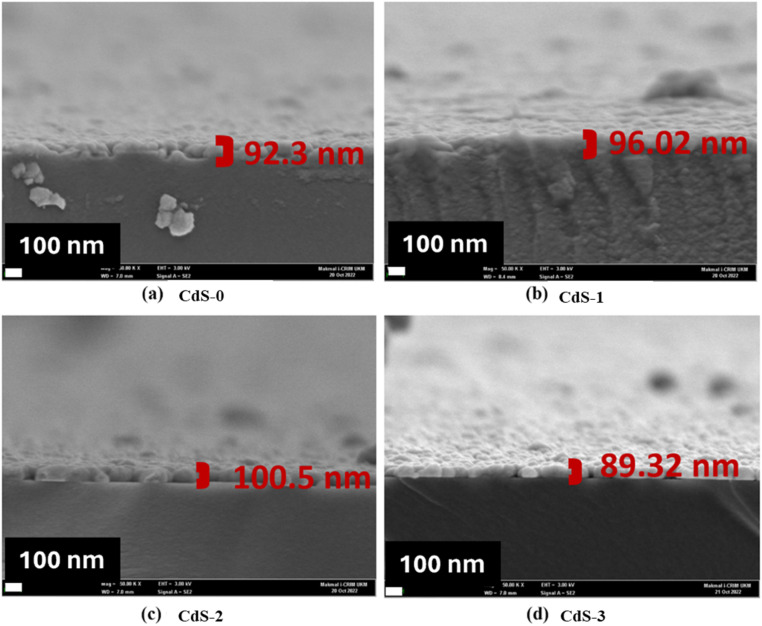
FESEM cross-sectional images of (a) CdS-0, (b) CdS-1, (c) CdS-2 and (d) CdS-3 films.

Fig. 5 shows the FESEM cross-sectional images of the deposited CdS films. From this figure, it was observed that the thickness of CdS films varied between 80 and 100 nm.

**Fig. 6:**
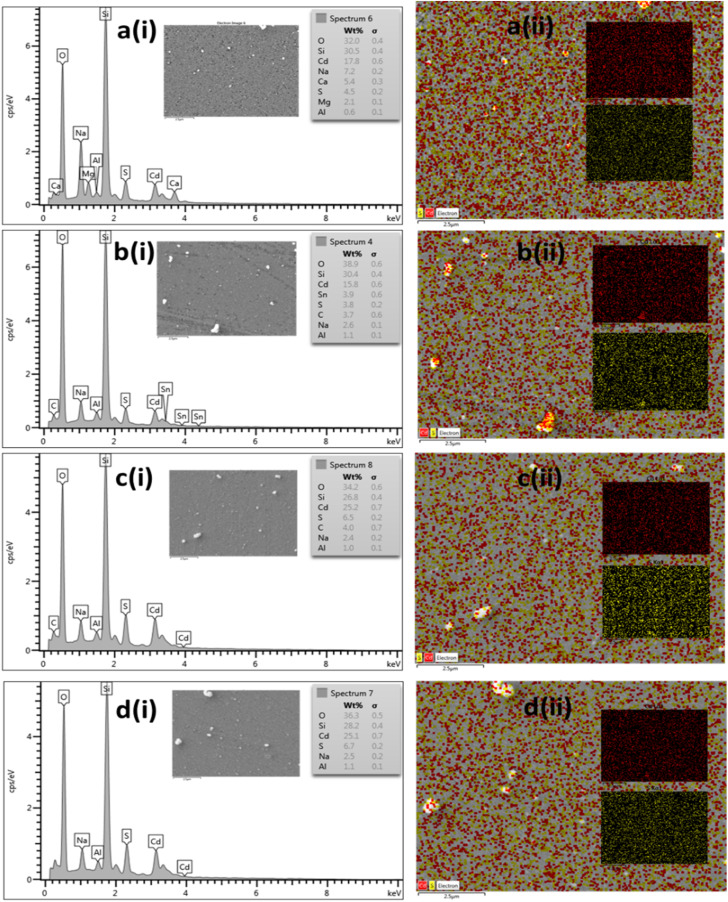
(i) EDX analysis and (ii) mapping of as-deposited CdS thin film with increasing concentration of (a) CdS-0, (b) CdS-1, (c) CdS-2, and (d) CdS-3.

The elemental compositions of the thin films were analyzed using EDX, as shown in Fig. 6. The EDX data, summarized in Table 3, reveal that the atomic ratio of Cd to S is approximately 1.1 for all deposited CdS thin films, indicating the formation of nano-crystalline, stoichiometric CdS films.^39^ Notably, CdS-1 exhibits a slightly higher atomic ratio of Cd/S (1.19), suggesting the presence of sulfur vacancies due to the coexistence of CdS with other cadmium compounds, which may also be intermediates or by-products of the CBD reaction.^40^ Additionally, EDX mapping in Fig. 6 confirms the uniform distribution of Cd and S across the films.

**Table 3:** Atomic ratio of Cd/S from EDX analysis

Sample	Elements	Atom. Conc. (%)	Atomic ratio (Cd/S)
CdS-0	Cd	53%	1.13
	S	47%	
CdS-1	Cd	54.3%	1.19
	S	45.7%	
CdS-2	Cd	52.5%	1.11
	S	47.5%	
CdS-3	Cd	51.7%	1.07
	S	48.3%	

### 3.4. Optical properties

**Fig. 7:**
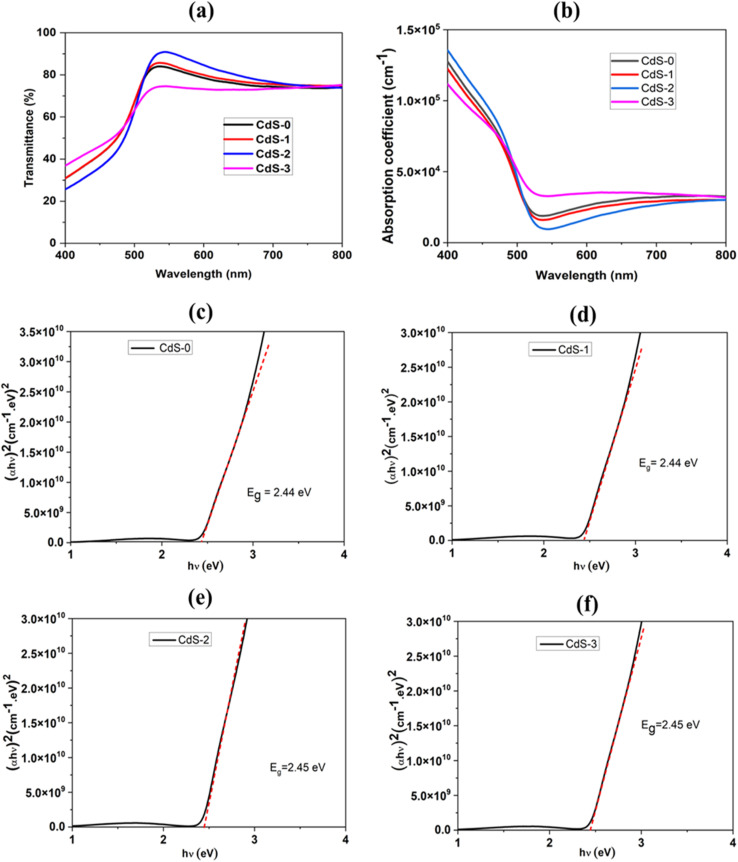
(a) Optical transmittance spectra, (b) absorption co-efficient spectra and Tauc plot of: (c) CdS-0, (d) CdS-1, (e) CdS-2, and (f) CdS-3.

Optical transmittance (*T*) and absorbance (*A*) plays a major factor in exploring any optoelectronic material. UV-vis spectroscopy-derived transmittance spectra of the different CdS thin films deposited with different ionic liquid concentrations are shown in Fig. 7(a).

The results showed that optical transmittance tends to vary between 75% and 90% within the visible light spectrum of 450–650 nm for different concentrations of ionic liquid. Absorption edges matched with the fundamental absorption edge for solution-deposited CdS thin films found in other studies.^41^ With the increment of the concentration of ionic liquid, the film transparency was increased, and it was improved up to 90% which was found for the film deposited with 0.069 M ionic liquid. This improvement in transparency may be attributed to the larger crystallite size of that film than other films, as Khimani *et al.* previously reported that the optical transmittance increased with the enlargement of crystallite size.^41,42^

From the absorbance values (*A*) of the films for different wavelengths of light (*λ*), absorption coefficients (*α*) were derived using the equation mentioned below^43,44^ – 7
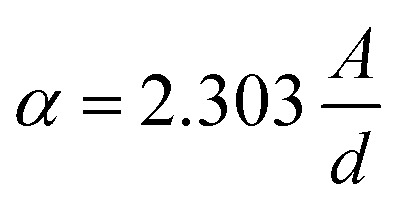
where, *d* = thickness of the film. Fig. 6b shows the absorption coefficient spectra for all CdS films. As the optical bandgap (*E*_g_) is a very important parameter for determining the photosensitivity range of a material, *E*_g_ for all deposited CdS films was determined from the Tauc plot that was plotted by linearizing the Tauc's equation as below:^45–48^

8 (*αhv*)^2^ = (*hv* − *E*_g_)

where, *hv* = photon energy. The Tauc plots for the CdS thin films are displayed in Fig. 7(c–f). Table 4 lists the computed bandgap values, which range from 2.44 eV to 2.45 eV. These consistent values show that the bandgap was unaffected by the ionic liquid. Instrumental accuracy and curve-fitting during the Tauc plot analysis are the causes of the experimental uncertainty, which is roughly ±0.01 eV. This small variation is within the uncertainty range, suggesting it is due to measurement errors rather than changes in material properties.

**Table 4:** Urbach and energy bandgap values for as-deposited CdS

Sample	Film thickness (nm)	Urbach energy (meV)	Bandgap (eV)
CdS-0	92.30	174.83	2.44
CdS-1	96.02	168.07	2.44
CdS-2	100.51	115.88	2.45
CdS-3	89.32	331.13	2.45

Urbach energy (*E*_u_), an important parameter representing the disorder, is often interpreted as the tail width of localized states in the bandgap.^49^*E*_u_ is constant or weakly dependent on temperature. It is assumed that the spectral dependence of the absorption edge in the low photon energy range follows the empirical Urbach rule given by.^15,16^

9 *α*(*ν*) = *α*_o_ exp(*hν*/*E*_u_)

where *α*_o_ is a constant. Calculated *E*_u_ for all deposited CdS films are tabulated in Table 4.

### 3.5. Electrical properties

Electron mobility (*μ*_e_), carrier concentration (*η*), and resistivity (*ρ*) values of as-deposited CdS thin films were obtained by the Hall effect measurement system, which is summarized in Table 5. The following equation represents the relationship between *η*, *μ*_e_ and *ρ*:^50^10
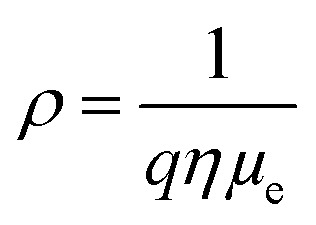
where *q* = electrical charge.

**Table 5:** Electrical properties of as-deposited CdS films

Sample name	Carrier concentration (cm^−3^)	Electron mobility (cm^2^ V^−1^ s^−1^)	Resistivity (Ω cm)
CdS-0	1.92 × 10^14^	16.1	1.37 × 10^4^
CdS-1	2.69 × 10^14^	4.84	5.28 × 10^3^
CdS-2	7.51 × 10^14^	16.5	1.85 × 10^3^
CdS-3	4.26 × 10^14^	3.15	5.32 × 10^3^

It has been observed that the charge carrier concentration value gradually increases with the increment of IL concentration into CdS thin films (CdS-1, CdS-2) up to 0.069 M. Beyond this point, it starts to decrease. The values are varied from 1.92 × 10^14^ cm^−3^ to 7.51 × 10^14^ cm^−3^. Moreover, it is also observed that the addition of IL reduces the resistivity of the CdS thin films by one order of magnitude. The CdS thin film with 0.069 M IL (CdS-2) exhibits the lowest resistivity and the highest mobility of 1.85 × 10^3^ Ω cm and 16.5 (cm^2^ V^−1^ s^−1^), respectively which indicates a better electrical property in comparison to others. These optimum electrical properties of the CdS-2 film can be correlated with the structural and morphological analysis of that film found from the Raman spectrum and FESEM images, respectively where CdS-2 film depicted moderate crystallinity and larger grain size without any pinholes and agglomeration. Moreover, the lowest Urbach energy of CdS-2 film representing fewer disorders or defects may be another possible reason for improved electrical properties.

### 3.6. Numerical simulation

**Fig. 8:**
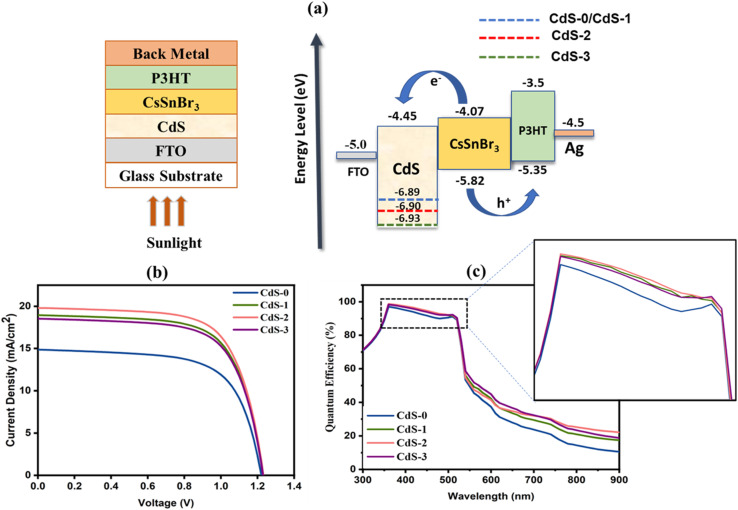
(a) Schematic illustration and band alignment of proposed PSC structure; (b) *J*–*V* characteristics curve and (c) quantum efficiency curve of proposed PSCs.

The electron transport layer (ETL) layer is mostly responsible for promoting the overall charge extraction capacity and tuning the crystallinity for better device performance in perovskite solar cells (PSCs).^51^ For evaluating the photovoltaic performances of the proposed PSC by employing IL-introduced CdS thin film as an electrical transport layer, a simulation study was performed. SCAPS-1D software was used for performing this computational study with a standard spectrum of AM1.5G (1000 W m^−2^; *T* = 300 K) for illumination. Here, deposited CdS thin films with different IL concentrations from 0 to 0.118 M were used as ETL. For the absorber and hole transport layer (HTL), CsSnBr_3_ and P3HT were employed with a band gap of 1.75 eV and 1.85 eV, respectively. Fig. 8(a) depicts the suggested device architecture (FTO/CdS/CsSnBr_3_/P3HT/Ag) with energy band alignment. The simulation parameters have been summarized in Table 6.

**Table 6:** Parameters used in device simulation

Parameters	FTO^25^	CdS-0 (ref. 52 and 53)	CdS-1 (ref. 52 and 53)	CdS-2 (ref. 52 and 53)	CdS-3 (ref. 52 and 53)	CsSnBr_3_ (ref. 54)	P3HT^55,56^
*d* (nm)	200	92.30 (exp.)	96.02 (exp.)	100.5 (exp.)	89.32 (exp.)	450	50
*E*_g_ (eV)	3.5	2.44 (exp.)	2.44 (exp.)	2.45 (exp.)	2.45 (exp.)	1.75	1.85
*χ* (eV)	4	4.45	4.45	4.45	4.45	4.07	3.5
*ε*_r_	9	10	10	10	10	5.9	3.4
*N*_C_ (cm^−3^)	2.2 × 10^17^	2.2 × 10^18^	2.2 × 10^18^	2.2 × 10^18^	2.2 × 10^18^	1 × 10^18^	1 × 10^22^
*N*_V_ (cm^−3^)	2.2 × 10^16^	1.9 × 10^19^	1.9 × 10^19^	1.9 × 10^19^	1.9 × 10^19^	1 × 10^18^	1 × 10^22^
*V*_th_ e^−^ (cm s^−1^)	1 × 10^7^	1 × 10^7^	1 × 10^7^	1 × 10^7^	1 × 10^7^	1 × 10^7^	1 × 10^7^
*V*_th_ p (cm s^−1^)	1 × 10^7^	1 × 10^7^	1 × 10^7^	1 × 10^7^	1 × 10^7^	1 × 10^7^	1 × 10^7^
*μ*_e_ (cm^2^ V^−1^ s^−1^)	20	16.1(exp.)	4.84 (exp.)	16.5 (exp.)	3.15 (exp.)	1 × 10^−1^	1 × 10^−4^
*μ*_p_ (cm^2^ V^−1^ s^−1^)	10	4.025	1.21	4.125	0.787	1 × 10^−1^	1 × 10^−3^
*N*_A_ (cm^−3^)	—	—	—	—	—	7 × 10^16^	3.17 × 10^13^
*N*_D_ (cm^−3^)	1 × 10^20^	1.92 × 10^14^ (exp.)	2.69 × 10^14^ (exp.)	7.51 × 10^14^ (exp.)	4.26 × 10^14^ (exp.)	—	—
*N*_t_ (cm^−3^)	—	—	—	—	—	1 × 10^14^	—

Fig. 8(b) and (c) illustrate the *JV* characteristics and quantum efficiency (QE) curve, respectively. From the *J*–*V* curve, it is observed that the deposited IL-introduced CdS thin films highly influenced the PSC performances. The device performances of the proposed PSC were increased with the increment of IL concentration into CdS thin films. However, the addition of IL above 0.069 M in CdS film preparation changes the film properties in such a way that the complete PSC performances started to decrease. This may be due to the decrement in charge carrier mobilities and thickness of the deposited IL-assisted CdS thin film. The highest efficiency of 16.57% was recorded for the PSC with CdS-2 (IL of 0.069 M) thin film, whereas *J*_sc_, *V*_oc_ and FF were found to be 19.80 mA cm^−2^, 1.23 V and 67.93%, respectively. Also, a better quantum efficiency (above 90%) was observed for PSC with CdS-2 film compared to others at the visible spectrum region ranging from 350 nm to 520 nm as shown in Fig. 8(d). The device performances are summarized in Table 7.

**Table 7:** Simulated perovskite solar cell performances

Device structure	*J*_sc_ (mA cm^−2^)	*V*_oc_ (V)	FF (%)	PCE (%)
FTO/CdS-0/CsSnBr_3_/P3HT/Ag	14.86	1.22	66.08	11.99
FTO/CdS-1/CsSnBr_3_/P3HT/Ag	18.95	1.23	67.72	15.79
FTO/CdS-2/CsSnBr_3_/P3HT/Ag	19.80	1.23	67.93	16.57
FTO/CdS-3/CsSnBr_3_/P3HT/Ag	18.52	1.23	67.60	15.40

## 4. Conclusions

CdS thin films were deposited on a glass substrate *via* the CBD technique with the addition of different concentrations of BMIMBF_4_ ionic liquid to analyze the impact. X-ray diffraction analysis revealed that the sharpness and intensity of the peak increased with the increment of ionic liquid concentration during the film preparation confirming the improvement of crystallinity in the films. From morphological studies, it was be concluded that the films exhibited better coverage with homogeneity and lesser pinholes, due to the use of ionic liquid, even when a lower concentration was used. Films showed good transparency with minor changes in bandgap ranging from 2.44 to 2.45 eV. The experimental uncertainty associated with these measurements is ±0.01 eV, indicating that the observed variations are within the range of experimental accuracy. The effect of morphological improvement of the CdS thin film with IL was also translated into good electrical properties. Analyzing the electrical properties, deposited CdS with the presence of 0.069 M IL was confirmed as the optimum one for which Raman analysis and surface morphology were found the most promising. That film performed as the best ETL in simulated PSC through SCAPS-1D simulation depicting the efficiency of 16.5%, that ensured the promising use of IL-assisted CdS film in photovoltaic application.

## Data availability

Data are available on request from the corresponding author.

## Conflicts of interest

There are no conflicts to declare.
